# Lack of association between use of efavirenz and death from suicide: evidence from the D:A:D study

**DOI:** 10.7448/IAS.17.4.19512

**Published:** 2014-11-02

**Authors:** Colette Smith, Lene Ryom, Antonella d'Arminio Monforte, Peter Reiss, Amanda Mocroft, Wafaa El-Sadr, Rainer Weber, Matthew Law, Caroline Sabin, Jens Lundgren

**Affiliations:** 1 Infection and Population Health, University College London, London, UK; 2 CHIP, University of Copenhagen, Copenhagen, Denmark; 3 Health Sciences, San Paolo University Hospital, Milan, Italy; 4 Academic Medical Center, University of Amsterdam and Stichting HIV Monitoring, Amsterdam, Netherlands; 5 Mailman School of Public Health, Columbia University, New York, USA; 6 Division of Infectious Diseases, University Hospital Zurich, Zurich, Switzerland; 7 Kirby Institute, University of New South Wales, Sydney, Australia

## Abstract

**Introduction:**

A recent meta-analysis of 4 RCTs showed an increased rate of suicidality events (suicidal ideation or attempted/completed suicide) associated with efavirenz (EFV) compared to other regimens, but only a trend towards a higher rate of completed/attempted suicides, as only 17 events occurred. We investigated the association between EFV use and completed suicide.

**Materials and Methods:**

All D:A:D participants were followed from study entry to the first of death, last study visit or 1 February 2013. Deaths are centrally validated using cause of death methodology, which assigns underlying, immediate and up to four contributing causes of death. Two endpoints were considered: 1) suicide or psychiatric disease as the underlying cause, and 2) suicide or psychiatric disease mentioned as an underlying, immediate or contributing cause of death (anywhere). Adjusted rate ratios were calculated using Poisson regression.

**Results:**

A total of 4420 deaths occurred in 49,717 people over 371,333 person-years (PY) (rate 11.9 per 1000 PY; 95% CI 11.6–12.3). A total of 193 deaths (rate 0.52; 0.45–0.59) had an underlying cause of suicide or psychiatric disease, and 482 deaths (1.30; 1.18–1.41) had suicide or psychiatric disease mentioned anywhere. A strong association with current CD4 count was seen: for suicide or psychiatric disease mentioned anywhere, rates were: 3.18 (2.55–3.80) for <200 cells/uL, 1.60 (1.29–1.90) for 201–350 cells/uL, 1.07 (0.86–1.29) for 351–500 cells/uL, 0.95 (0.80–1.09) for >500 cells/uL and 1.30 (1.18–1.41) for unknown. Highest rate of suicide or psychiatric deaths were seen in ART-experienced people currently off ART, but no differences were seen according to current ART regimen, which remained after adjustment (Table 1). Consistent results were obtained when considering additional endpoints of suicide alone as the underlying cause and death from suicide or any possibly related cause (psychiatric disease, drug overdose, alcohol related, accidental or violent), as well as considering recent EFV use in the previous 3 and 6 months.

**Conclusions:**

The finding of no higher death rates from suicide amongst those receiving EFV is reassuring. However, there is likely confounding by indication in our observational study. In light of conflicting results from RCTs, this potentially could suggest that in clinical practice EFV may be less frequently prescribed in those with underlying psychiatric conditions.

**Table 1:** Rates of death from suicide, according to current ART use

	Number/person-years	Rate per 1000 person-years (95% CI)	Adjusted RR (95% CI)	*p*
Suicide or psychiatric disease as underlying cause of death				
Total	193/371,333	0.52 (0.45–0.59)		
EFV-containing	24/78,580	0.31 (0.81–0.43)	0.59 (0.33–1.06)	<0.0001
Other NNRTI-containing	31/64,288	0.48 (0.31–0.65)	0.93 (0.53–1.62)	
Other ART	66/157,664	0.42 (0.32–0.52)	0.81 (0.49–1.32)	
No ART–naïve	21/40,454	0.52 (0.30–0.74)	1.00	
No ART–experienced	51/30,348	1.68 (1.22–2.14)	3.24 (1.95–5.38)	
Suicide of psychiatric disease, mentioned as underlying, immediate of contributing cause of death				
Total	482/371,333	1.30 (1.18–1.41)		
EFV-containing	60/78,580	0.76 (0.57–0.96)	0.42 (0.28–0.63)	<0.0001
Other NNRTI-containing	72/64,288	1.12 (0.86–1.38)	0.68 (0.46–1.00)	
Other ART	162/157,664	1.03 (0.87–1.19)	0.52 (0.37–0.73)	
No ART–naïve	62/40,454	1.53 (1.15–1.91)	1.00	
No ART–experienced	126/30,348	4.15 (3.43–4.88)	2.29 (1.63–3.21)	

